# Adenoid cystic carcinoma of buccal mucosa: A report of two rare cases and review of literature

**DOI:** 10.1002/ccr3.3375

**Published:** 2020-11-21

**Authors:** Abbas Karimi, Alireza Parhiz, Negin Eslamiamirabadi, Monir Moradzadeh Khiavi, Samira Derakhshan

**Affiliations:** ^1^ Oral and Maxillofacial Surgery Department Craniomaxillofacial Research Center Shariati Hospital Tehran University of Medical Sciences Tehran Iran; ^2^ Oral and Maxillofacial Surgery Department Craniomaxillofacial Research Center Sina Hospital Tehran University of Medical Sciences Tehran Iran; ^3^ MSc of Dental Sciences Faculty of Dentistry McGill University Montreal QC Canada; ^4^ Oral and Maxillofacial Pathology Department School of Dentistry Tehran University of Medical Sciences Tehran Iran

**Keywords:** adenoid cystic, buccal mucosa, carcinoma, oral

## Abstract

In the oral cavity, adenoid cystic carcinomas of the buccal mucosa are extremely rare. Minor salivary grand adenoid cystic carcinoma should receive aggressive treatment to achieve negative surgical margins to inhibit recurrence.

## Graphical abstract

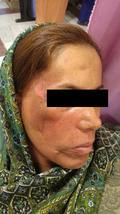

## 1 INTRODUCTION

Adenoid cystic carcinoma (AdCC) is a malignant tumor mostly occurring in the head and neck salivary glands. In this article, two rare cases of adenoid cystic carcinoma occurring in the buccal mucosa with their treatment and long‐term follow‐up are presented.

Adenoid cystic carcinoma (AdCC) was initially described as "cylindroma" in 1856 by Billroth, as it contained long amorphous compartments named "cylinders" in its histological view. Later on, the term “adenoid cystic carcinoma”(AdCC) replaced the term cylindroma for defining this tumor.^1^ AdCC accounts for 10% of salivary gland tumors and for approximately 1% of all malignancies of the head and neck.^2^ AdCC arises more frequently in minor salivary glands, in comparison with major salivary glands.^3^ AdCC of minor salivary glands is believed to have a worse prognosis, compared to those of the major salivary glands. Pain can be a paramount symptom of the disease, due to the tumor's proneness toward perineural invasion.^4^ Although this tumor is likely to occur at almost any age, it is most commonly observed in women, in the 5th and 6th decades of life.^5^ In a recent study, the AdCC occurrence rate in women and men was 60:40, respectively.^6^

AdCC often manifests itself as a small and slowly growing tumor. However, it is diagnosed at an advanced stage in the most cases.^7^ It grows with a slower rate, in comparison with other carcinomas, and has a low prevalence of spreading into local and regional lymph nodes. Nevertheless, local and distant recurrences and also hematogenous spread are relatively common.^3^ Distant metastasis is quite common, with the highest prevalence in the lungs, followed by bones, liver, and brain.^8^

In this article, two cases of adenoid cystic carcinoma which occurred in the right buccal mucosa are presented.

## 2 CASE PRESENTATION

### 2.1 Case No. 1

**Figure 1:**
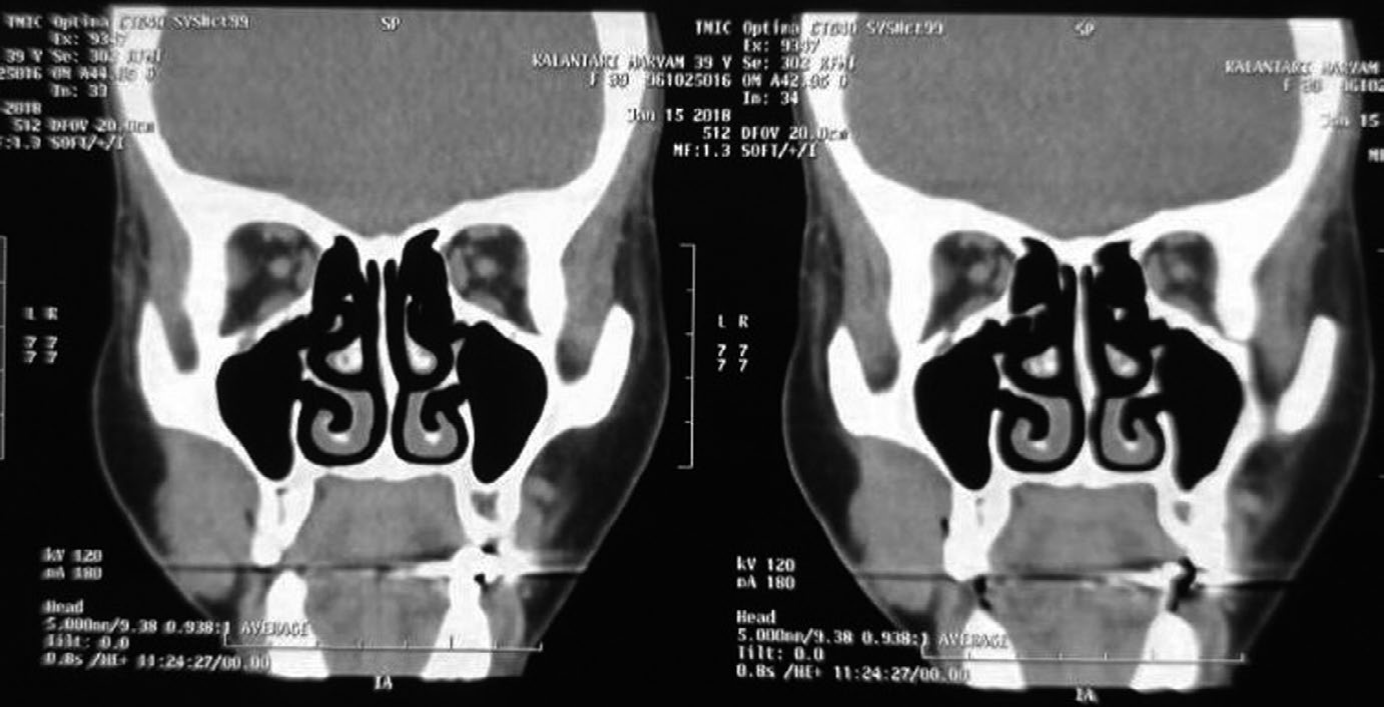
CT scan view of the lesion demonstrates ill‐defined mass of right buccal area with invasion to the surrounding structures of the skin and zygomatic bone

**Figure 2:**
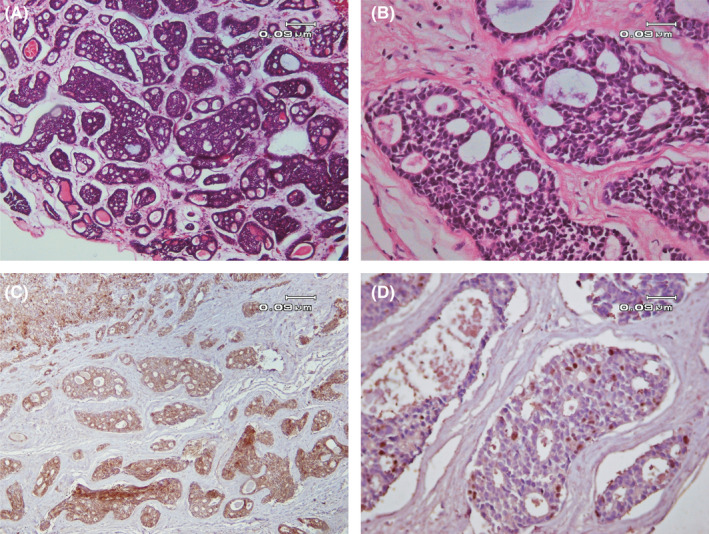
Histopathologic views of the lesion. A, Small basaloid tumoral cells in various sized nests (H&E staining, ×100 magnification). B, Classic cribriform pattern in tumoral cells (H&E staining, ×100 magnification). C, Immunohistochemistry results show severe diffuse positive immunoreaction for C‐kit (×100 magnification). D, ki67 was positive in about 10% of tumoral cells

A 39‐year‐old woman was referred to the Department of Oral and Maxillofacial Surgery with the chief complaint of a mass in the right buccal mucosa, which had been formed more than 2 months ago. The patient was also suffering from a severe trismus due to the lesion. In the intraoral examination, an ulcerative sessile painless exophytic mass in the right buccal mucosa was observed. Lymphadenopathy was not detected. No past medical or allergic history was found. Computed tomography scan (CT scan) of the patient showed large mass in right buccal mucosa which was attached to superficial skin and also due to bone depression in right zygomatic bone (Figure 1). Incisional biopsy was carried out. The histopathologic examination revealed a neoplasm composed of small hyperchromatic basaloid cells arranged mostly in cribriform, and occasionally in solid and tubular patterns within a fibromyxoid stroma. The cyst‐like spaces among the tumoral cells contained eosinophilic or basophilic material. The tubular pattern consisted of small ducts lined by several cuboidal cells which contained hyalinized material (Figure 2). Nuclear pleomorphism, atypia, and mitotic activity were very low. These histomorphologic features were in favor of adenoid cystic carcinoma. Immunohistochemistry (IHC) was performed to confirm the diagnosis and to rule out other adenocarcinomas containing basaloid cells. C‐kit antigen was diffusely positive in the tumoral cells. P63 was positive and scattered. Proliferation activity was also evaluated. Ki67 was positive in about 10% of the tumoral cells (Figure 2).

These results were compatible with adenoid cystic carcinoma with a salivary gland origin. The patient underwent examinations to rule out distant metastasis by computed tomography of the chest and abdominal areas. Fortunately, no evidence of distant metastasis was observed.

**Figure 3:**
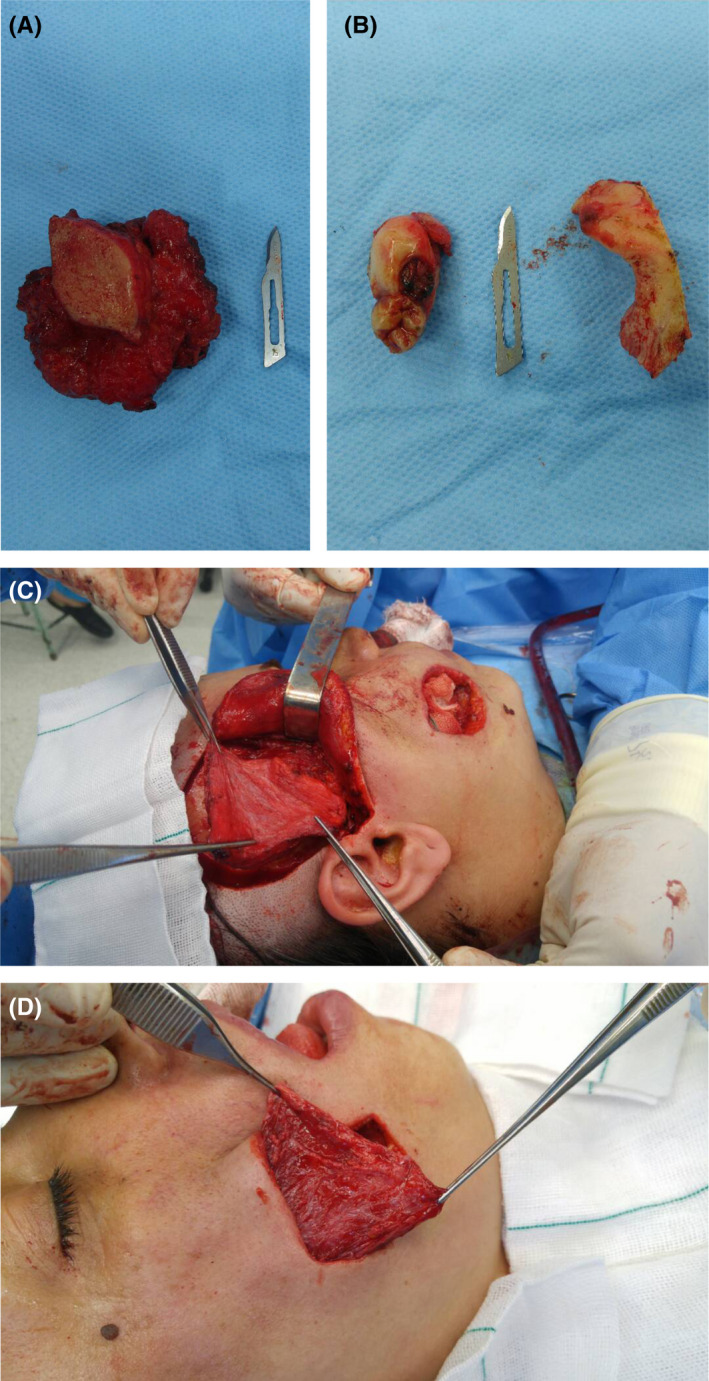
Surgical intervention. A, Whole excited lesion with safe margin. B, Excited zygomatic bone and maxillary tuberosity. C, Pedicled right temporalis muscle flap was used for reconstruction of the surgical area. D, Transferred flap to the surgical area

**Figure 4:**
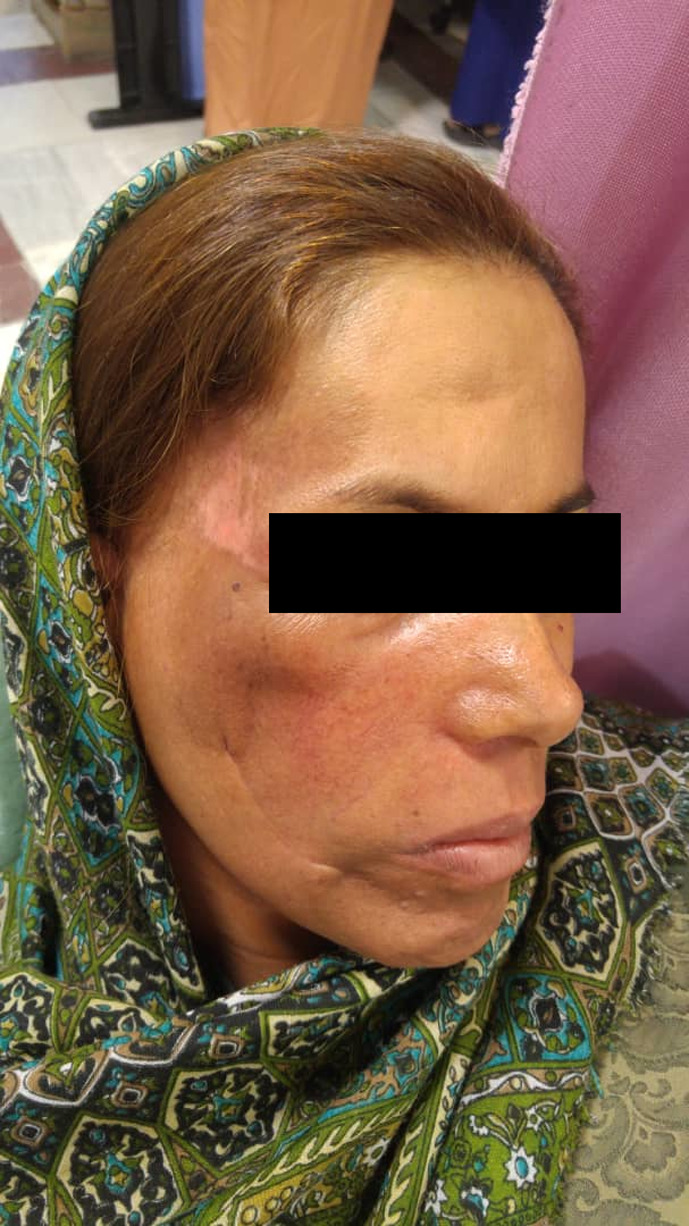
Follow‐up of the patient after one and half year shows ideal healing without significant problem

The lesion was excised completely, and reconstruction of the surgical area was done by pedicle temporalis muscle flap (Figure 3). Adjuvant radiotherapy was also carried out. After 24 months of follow‐up, the patient is alive without any noticeable problems (Figure 4).

### 2.2 Case No. 2

**Figure 5:**
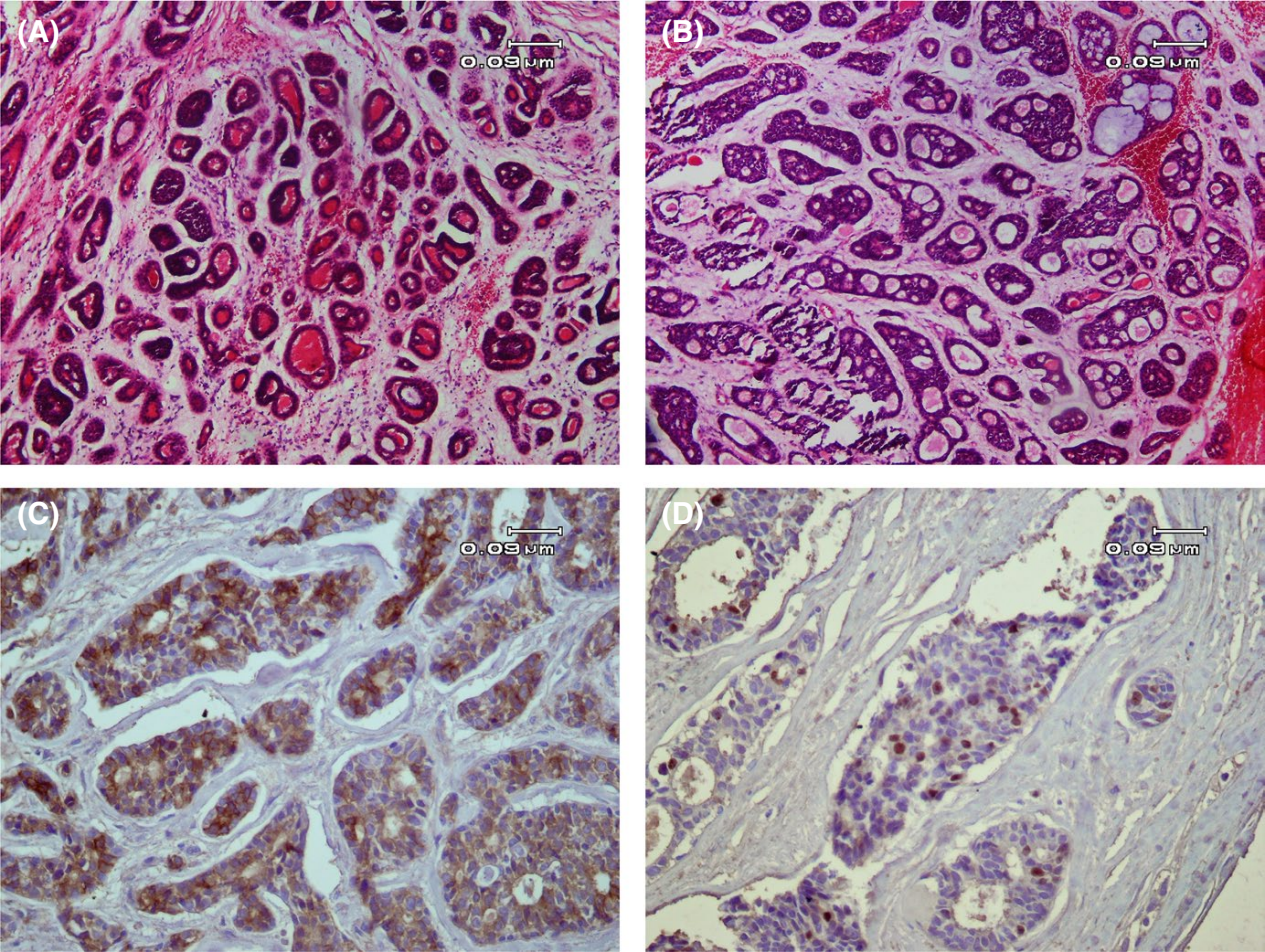
A, Microscopic examinations show tubular pattern of small basaloid tumoral cells (H&E staining, ×100 magnification). B, Tumoral cells demonstrate admixture of cribriform and tubular pattern (H&E staining, ×100 magnification). C, Diffuse positive C‐kit immunoreaction in tumoral cells (IHC, ×400 magnification). D, Positive immunoreaction for ki67 in about 10% of tumoral cells (IHC, ×400 magnification)

A 37‐year‐old man was presented to the Department of Oral and Maxillofacial Surgery with a chief complaint of a mass in the buccal mucosa of the left side. Intraoral examination revealed a pink submucosal lesion with duration of 8 months. No noticeable past medical and dental history was detected. There was so sign of lymphadenopathy in extraoral examination. Incisional biopsy was conducted. The histopathologic examination indicated a neoplastic lesion composed of islands of myoepithelial and ductal cells that were arranged in the tubular pattern in some areas. The tumor cells were small and cuboidal exhibiting basophilic nuclei and scant cytoplasm with minimal atypia and mitotic figures. The IHC findings were compatible with adenoid cystic carcinoma (Figure 5).

The lesion was surgically excised, and postoperative radiotherapy was performed for the patient. The patient situation after the treatment was different from the case number one. Trismus was the most important complication after treatment due to surgery and radiotherapy. No distant metastasis was eventually detected in the patient's workups every three months during 2 years after treatment.

## 3 DISCUSSION

The World Health Organization (WHO) has defined AdCC as "a basaloid tumor containing epithelial and myoepithelial cells in diverse morphological configurations, such as tubular, cribriform, and solid patterns. Its clinical course is relentless and usually has a fatal outcome".^9^

A review of literature was carried out in this study, which is demonstrated in Table 1.

**Table 1:** Literature review of the reported cases of\ adenoid cystic carcinoma of the buccal mucosa (2010 ‐present)

	Age and sex	Pain	Duration	Consistency	Paraclinical auxiliary examinations	Size	AdCC subtype	Perineural invasion	Metastasis	Lymph node involvement	Treatment	Follow‐up
Singh^10^ (2010)	50/M	Yes	1.5 y	Firm	–	3 cm	Cribriform	–	Negative	–	Excision under general anesthesia	Till date(for 3 y)
Ajila & Hegde^11^ (2012)	45/F	Yes (mild and continuous)	3 mo	Soft to firm	Panoramic radiography Ultrasonography	1 × 1 cm	Cribriform	Yes	–	–	Excision without adjuvant therapy	3 y
Kumar^12^ (2013)	26/F	Yes (in palpation)	1 y	Hard	–	3cm	Cribriform	–	Negative	Negative	Complete surgical removal	Till date
Naik^13^ (2013)	48/M	Yes	1 month	Firm	Conventional radiography Ultrasonography MRI	3 × 3×2 cm	Cribriform	Yes (& intraneural invasion)	–	–	Excision without adjuvant therapy	10 y
Vidyalakhshmi^14^ (2014)	48/F	No	1 y	Firm	Panoramic radiography	2 × 3 cm	Cribriform	–	–	–	Excision and referral to oncologist	6 mo
Garg^15^ (2016)	40/F	Yes	1 y	Firm	–	1.5 × 1 cm	Solid (with few areas of cribriform and tubular)	–	–	–	Excision under local anesthesia	Till date
Dalirsani^16^ (2016)	47/M	Yes (in eating and palpation)	3 y	Firm	Sonography CT scan	3 cm	Cribriform	–	Positive	Negative	Excision, radiotherapy, and chemotherapy	Till date
Bansal^8^ (2016)	5/M	–	9 y	–	–	–	–	–	Yes	–	Excision, neck dissection, radiotherapy, and chemotherapy, referral to the Department of Oncology	–
Kumar^17^ (2018)	16/M	Yes	4 mo	Firm	Sialadenoscopy Sialography face and neck MRI	1.08 × 0.7× 1.13 cm	Cribriform	Yes	Negative	Negative	Excision, radiotherapy	1 y
Venkatesh^18^ (2018) (case 1)	70/M	–	–	–	–	2 × 1×1 cm	Solid	No	–	–	Excision	–
Venkatesh^18^ (2018) (case 2)	60/M	–	–	–	–	0.5 × 0.2 cm	Solid	–	–	–	–	–

Eleven cases of AdCC in the buccal mucosa have been reported via articles thus far. In this article, two other cases of AdCC in the buccal mucosa have been reported. The first case was a 39‐year‐old woman with a painless exophytic mass in the buccal mucosa for more than two months. The second case was a 37‐year‐old man with a mass in the left buccal mucosa since 8 months.

In Vidyalakshmi et al's case, the swelling had arisen after a left upper posterior tooth was extracted. Furthermore, a single submandibular lymph node on the left side of the patient's face was palpable, a phenomenon rarely occurring in adenoid cystic carcinoma. The lymph node was firm, mobile, nontender, and had a size of less than 1 cm.^14^

The patient in Dalirsani et al's study had a leukoedema accompanying the AdCC lesion on the same side. Superficial ulceration was evident in the aforementioned tumoral lesion, unlike the reported cases in this article, which were free of ulceration. At the time, a stage I diagnosis (T_2_N_0_M_0_) was made, and the patient underwent 35 cycles of radiotherapy and chemotherapy. Two years later, the patient was once again referred to the department, with a chief complaint of a 1.5 cm firm mass on his right frontal region. Following an incisional biopsy, a diagnosis of adenoid cystic carcinoma was established, and the patient was referred for chemotherapy and further investigations to an oncologist. Following a CT scan, a stage IV lung metastasis was diagnosed. The patient underwent 3 cycles of cisplatin and 5‐fluorouracil chemotherapy, which were ineffective in reducing the tumor's size. Therefore, 3 more cycles were carried out with taxol and carboplatin. The patient remained under oncologists' supervision until date.^16^

In the case reported by Bansal et al, the patient who had received treatment for adenoid cystic carcinoma of the buccal mucosa in 2005 was referred again, with a chief complaint of coughs for 4 months, breathlessness for the last 2 months, and fever for 20 days. A CECT scan view of thorax and upper abdomen identified parenchymal metastatic deposits. Multiple osseous metastatic deposits and a few small mediastinal lymph nodes were observed. Fine needle aspiration cytology (FNAC) confirmed that the metastatic deposits belonged to the adenoid cystic carcinoma, which the patient was diagnosed in 2005. The patient was referred to the Department of Oncology for further management.^8^ In Kumar et al's study (2018), brain MRI and chest X‐ray revealed no evidence of distant metastases.^17^

There are three histopathological views for adenoid cystic carcinoma: cribriform, tubular, and solid patterns. The cribriform pattern, the most common, has a view of islands of basaloid cells, surrounded by cyst‐like spaces in different sizes, forming a "Swiss cheese" pattern. The tubular histological subtype has a closely similar display, but with cells arranged and organized in nests surrounded by different amounts of often hyalinized eosinophilic stroma. The solid subtype manifests aggregates of basaloid cells without tubular or pseudocystic formations.^3^Since polymorphism is a common phenomenon in AdCC, it is possible to see all three aforementioned patterns in one specimen. Therefore, MD Anderson introduced a pathological grading system, to which is now contributed worldwide ^19^:

Grade I: tubular and cribriform together, without a solid pattern.
Grade II: mostly cribriform, with less than 30% of solid pattern.
Grade III: solid being the predominant subtype.

The differential diagnosis includes polymorphous low‐grade adenocarcinoma (PLGA), salivary duct carcinoma, and basaloid squamous cell carcinoma.^18^ PLGA demonstrates large tumoral cells than AdCC with vesicular nuclei versus small basaloid cells of AdCC. Single file appearance can also help to distinguish PLGA from AdCC. Salivary duct carcinoma (SDC) does not show cribriform pattern of AdCC and PLGA. On the other hand, SDC is a high‐grade carcinoma with prominent nuclear pleomorphism and atypical mitotic figures. Comedo‐type necrosis can help to diagnosis between SDC and other tumors of salivary glands.^20^ Basaloid squamous cell carcinoma (BSCC) is a high‐grade malignancy of keratinocytes which can mimic AdCC, histopathologically. Superficially squamous cell carcinoma, large eosinophilic cytoplasm of tumoral cells, and keratin pearls can help to distinguish between BSCC and AdCC. Ancillary studies such as immunohistochemistry examinations can help to definitive diagnosis.

Perineural invasion, sometimes associating adenoid cystic carcinoma, is defined as "a form of direct primary spread of neoplasm which may not necessarily be macroscopically continuous with the main focus of the tumor, but is usually microscopically continuous." The second and third branches of the trigeminal nerve are most frequently affected. The descending branches of the facial nerve and smaller cranial branches can also get involved. Perineural involvement is claimed to be an indicator of poor prognosis.^19^ Furthermore, it can increase the chances of recurrence. Recent data argue that intraneural invasion, rather than perineural, can have a higher impact on the survival rate in the AdCC of the head and neck.^3^

In Naik et al's case, slight perineural thickening of the facial nerve was observed. In a more precise examination, perineural and intraneural invasion were evident, due to which the patient had mild degrees of facial palsy.^13^

Contrary to other types of carcinomas, distant hematogenous metastases are much more common than regional lymph node metastases in AdCC. Hematogenous metastases in AdCC can remain asymptomatic for a considerable period of time, especially lung metastases, which apparently has a slow progress rate.^19^

Singh et al (2010) used paraclinical assessments, such as ultrasonography of upper abdomen, noncontrast proton MRI of the oral cavity, PA view of the chest, axial CT scan of the head and face, lateral neck radiograph, submentovertex view of the skull (35°), PA view of the pelvic girdle, and CT scan of chest, to rule out metastasis.^10^

The primary goal of treatment for patients affected with AdCC is local control of the tumor, normal function, and preventing distant metastases.^21^ Radical surgery with wide resection margins may not be sufficient per se, as achieving disease‐free margins can be difficult due to AdCC's propensity to perineural invasion and some lesions' challenging anatomical access.^2^ Radiation is noncompulsory for small tumors (T_1_N_0_) but must be considered for cases having low‐grade tumors in association with perineural invasion, or evidence of tumor seeding during surgery. A lower radiation dose has also been recommended for patients with tumors located in lymphatically rich areas.^22^ Using radiotherapy as primary treatment is proposed when surgery is not feasible.^3^ In addition, radiation can be a standard treatment for alleviating bone and brain metastases.^23^

As AdCC's sensitivity to chemotherapy is relatively low, systemic chemotherapy for AdCC remains controversial. On the other hand, chemotherapeutic treatment has proven to be effective in a rather low percentage of patients with recurrences or metastases. The first‐line chemotherapy choice is highly dependent on patients' comorbidities and characteristics.^2^

Tumors of the minor salivary glands can more easily infiltrate the surrounding extraglandular tissues, increasing dissemination of the tumor cells, and thus rendering resection with disease‐free margins more difficult. The cribriform variant is believed to have the best prognosis, while the solid pattern has the worst, with the tubular subtype having an intermediate‐level prognosis.^19^

## 4 CONCLUSION

Adenoid cystic carcinoma is a malignant tumor characterized by features such as slow growth, high infiltration potential, and hematogenous distant metastasis. Minor salivary glands AdCCs should receive aggressive treatment to achieve negative surgical margins to inhibit recurrence. In this article, two cases of adenoid cystic carcinomas having occurred in the buccal mucosa were reported. For both cases, long‐term follow‐ups were carried out, which are essential for monitoring signs of recurrence or distant metastasis.

## CONFLICT OF INTEREST

None declared.

## AUTHOR CONTRIBUTIONS

Abbas Karimi and Alireza Parhiz: involved in patient's treatment and follow‐up modalities. Negin Eslamiamirabadi: wrote the manuscript and collected the data. Monir Moradzadeh Khiavi: reviewed pathologic report. Samira Derakhshan: wrote the manuscript, reviewed pathologic report, and supervised the study.

## ETHICAL APPROVAL

Tehran University of Medical Sciences, School of Dentistry, Oral and Maxillofacial Pathology Department approved this report.

## PATIENT CONSENT

The authors of this article have obtained all required consent forms from both patients. Their consent has been given for their images and other clinical information to be reported in this article without including their names.
